# Supplementary material for: Auxiliary subunits keep AMPA receptors compact during activation and desensitization
Source: eLife. 2018 Dec 6;7:e40548. doi: 10.7554/eLife.40548 (PMC6324883; doi:10.7554/eLife.40548)
Supplement: Figure 2—source data 1. — The effect was measured after 1 min of exposure to a given bis-MTS reagent. [file elife-40548-fig2-data1.docx]

Figure 2 – source data 1. Statistics of trapping desensitized wild-type (WT), A665C and V666C receptors with different bis-MTS cross-linkers. The statistics in the Table accompanies data in Figure 2D-E. The effect was measured after 1 minute of exposure to a bis-MTS.

| Desensitizing receptors | | | | | | | |
| --- | --- | --- | --- | --- | --- | --- | --- |
| GluA2 WT | | | | | | | |
|  | M1M | M3M | bMTSp | M6M | M8M | M10M | w/o MTS |
| Active Fraction | 0.89 | 0.84 | 0.78 | 0.88 | 0.85 | 0.81 | 0.97 |
| SEM: | 0.08 | 0.06 | 0.08 | 0.07 | 0.05 | 0.05 | 0.03 |
| *n*: | 4 | 11 | 6 | 6 | 6 | 8 | 30 |
| GluA2 V666C | | | | | | | |
|  | M1M | M3M | bMTSp | M6M | M8M | M10M | w/o MTS |
| Active Fraction | 0.14 | 0.12 | 0.070 | 0.12 | 0.11 | 0.30 | 0.86 |
| SEM: | 0.02 | 0.02 | 0.01 | 0.02 | 0.03 | 0.03 | 0.02 |
| *n*: | 6 | 13 | 7 | 7 | 9 | 16 | 44 |
| *P* (vs. WT): | < 10^-7^ | < 10^-7^ | < 10^-7^ | 0.0004 | 0.0002 | < 10^-7^ | 0.003 |
| *P* vs. M10M: | 0.006 | < 10^-7^ | 0.0004 | 0.002 | 0.003 |  |  |
| *P* vs. bMTSp: | 0.03 | 0.09 |  | 0.2 |  |  |  |
| *P* vs. M8M: | 0.8 | 0.9 | 0.3 | 0.8 |  |  |  |
| *P* vs. M1M: |  | 0.7 |  | 0.3 |  |  |  |
| GluA2 A665C | | | | | | | |
|  | M1M | M3M | bMTSp | M6M | M8M | M10M | w/o MTS |
| Active Fraction | - | 0.45 | 0.48 | 0.53 | 0.48 | 0.40 | 0.62 |
| SEM: | - | 0.04 | 0.07 | 0.08 | 0.075 | 0.03 | 0.04 |
| *n*: | - | 4 | 7 | 0.01 | 8 | 5 | 23 |
| *P* (vs. WT): | - | 0.02 | 0.02 | 7 | 0.006 | 0.003 | < 10^-7^ |
