# Supplementary material for: Auxiliary subunits keep AMPA receptors compact during activation and desensitization
Source: eLife. 2018 Dec 6;7:e40548. doi: 10.7554/eLife.40548 (PMC6324883; doi:10.7554/eLife.40548)
Supplement: Figure 5—source data 1. — The peak current reduction was measured after 1 min of exposure to a given bis-MTS reagent. [file elife-40548-fig5-data1.docx]

Figure 5 – source data 1. Statistics of trapping active V666C receptors with different bis-MTS cross-linkers. The statistics in the Table accompany data in Figure 5B. The peak current reduction was measured after 1 minute of exposure to a bis-MTS.

| Active receptors | | | | | | | |
| --- | --- | --- | --- | --- | --- | --- | --- |
| GluA2 V666C | | | | | | | |
|  | M1M | M3M | bMTSp | M6M | M8M | M10M | w/o MTS |
| Active Fraction | 0.51 | 0.38 | 0.27 | 0.31 | 0.24 | 0.46 | 0.84 |
| SEM: | 0.02 | 0.02 | 0.01 | 0.03 | 0.05 | 0.05 | 0.02 |
| *n*: | 12 | 9 | 9 | 8 | 9 | 12 | 59 |
| *P* (vs. w/o MTS): | < 10^-7^ | < 10^-7^ | < 10^-7^ | < 10^-7^ | < 10^-7^ | < 10^-7^ |  |
| *P* vs. M10M: | 0.4 | 0.2 | 0.0008 | 0.03 | 0.003 |  |  |
| *P* vs. bMTSp: | < 10^-7^ | < 10^-7^ |  | 0.3 |  |  |  |
| *P* vs. M8M: | 0.0004 | 0.01 | 0.6 | 0.3 |  |  |  |
| *P* vs. M1M: |  | 0.0004 |  | < 10^-7^ |  |  |  |
