# Supplementary material for: Auxiliary subunits keep AMPA receptors compact during activation and desensitization
Source: eLife. 2018 Dec 6;7:e40548. doi: 10.7554/eLife.40548 (PMC6324883; doi:10.7554/eLife.40548)
Supplement: Figure 6—source data 1. — The current reduction was measured after 1 min of exposure to a given bis-MTS reagent. [file elife-40548-fig6-data1.docx]

Figure 6 – source data 1. Statistics of trapping V666C receptors with Stargazin (Stg) with different bis-MTS cross-linkers. The statistics in the Table accompany data in Figure 6C. The current reduction was measured after 1 minute of exposure to a bis-MTS.

| Desensitized receptors with Stg | | | | | | | |
| --- | --- | --- | --- | --- | --- | --- | --- |
| GluA2 V666C + Stg | | | | | | | |
|  | M1M | M3M | bMTSp | M6M | M8M | M10M | w/o MTS |
| Active Fraction | 0.43 | 0.35 | 0.34 | 0.37 | 0.43 | 0.59 | 0.89 |
| SEM: | 0.02 | 0.04 | 0.03 | 0.02 | 0.01 | 0.04 | 0.02 |
| *n*: | 9 | 7 | 9 | 7 | 9 | 7 | 48 |
| *P* (vs. w/o MTS): | < 10^-7^ | < 10^-7^ | < 10^-7^ | < 10^-7^ | < 10^-7^ | < 10^-7^ | < 10^-7^ |
| *P* vs. M10M: | 0.006 | 0.005 | 0.0006 | 0.002 | 0.0006 |  |  |
| *P* vs. bMTSp: | 0.02 | 0.8 |  | 0.4 |  |  |  |
| *P* vs. M8M: | 0.9 | 0.06 | 0.009 | 0.04 |  |  |  |
| *P* vs. M1M: |  | 0.08 |  | 0.08 |  |  |  |
