# Supplementary material for: Auxiliary subunits keep AMPA receptors compact during activation and desensitization
Source: eLife. 2018 Dec 6;7:e40548. doi: 10.7554/eLife.40548 (PMC6324883; doi:10.7554/eLife.40548)
Supplement: Transparent reporting form [file elife-40548-transrepform.pdf]

eLIFE
1st Floor
24 Hills Road
Cambridge CB2 1JP, UK
P 01223 855340
W elifesciences.org
T @elife

eLife's transparent reporting form

We encourage authors to provide detailed information *within their submission* to facilitate the interpretation and replication of experiments. Authors can upload supporting documentation to indicate the use of appropriate reporting guidelines for health-related research (see EQUATOR Network), life science research (see the BioSharing Information Resource), or the ARRIVE guidelines for reporting work involving animal research. Where applicable, authors should refer to any relevant reporting standards documents in this form.

If you have any questions, please consult our Journal Policies and/or contact us: editorial@elifesciences.org.

Sample-size estimation

- You should state whether an appropriate sample size was computed when the study was being designed
- You should state the statistical method of sample size computation and any required assumptions
- If no explicit power analysis was used, you should describe how you decided what sample (replicate) size (number) to use

Please outline where this information can be found within the submission (e.g., sections or figure legends), or explain why this information doesn't apply to your submission:

We did not compute appropriate sample sizes but followed convention in the electrophysiology field to report at least 5-6 recordings for each condition where practical. This sample size is sufficient to detect effects in test experiments of ~10%, when experiments are done carefully.

Replicates

- You should report how often each experiment was performed
- You should include a definition of biological versus technical replication
- The data obtained should be provided and sufficient information should be provided to indicate the number of independent biological and/or technical replicates
- If you encountered any outliers, you should describe how these were handled
- Criteria for exclusion/inclusion of data should be clearly stated
- High-throughput sequence data should be uploaded before submission, with a private link for reviewers provided (these are available from both GEO and ArrayExpress)

Please outline where this information can be found within the submission (e.g., sections or figure legends), or explain why this information doesn't apply to your submission:

The number of patches is indicated in the text for each condition (*n*). Each patch was obtained from a different cell (technical replication). For each condition, patches were obtained from 3 or more different transfection experiments, using one or more different transfection dishes (biological replication), as described in the 'Analysis' section. When introduced, individual data points are plotted in each graph. For subsequent usage of these data, mean and standard deviation are shown. Exclusion criteria for patch clamp data can be found in the 'Analysis' section.

eLife Sciences Publications, Ltd is a limited liability non-profit non-stock corporation incorporated in the State of Delaware, USA, with company number 5030732, and is registered in the UK with company number FC030576 and branch number BR015634 at the address 1st Floor, 24 Hills Road, Cambridge CB2 1JP | August 2014

Statistical reporting

- Statistical analysis methods should be described and justified
- Raw data should be presented in figures whenever informative to do so (typically when N per group is less than 10)
- For each experiment, you should identify the statistical tests used, exact values of N, definitions of center, methods of multiple test correction, and dispersion and precision measures (e.g., mean, median, SD, SEM, confidence intervals; and, for the major substantive results, a measure of effect size (e.g., Pearson's r, Cohen's d)
- Report exact p-values wherever possible alongside the summary statistics and 95% confidence intervals. These should be reported for all key questions and not only when the p-value is less than 0.05.

Please outline where this information can be found within the submission (e.g., sections or figure legends), or explain why this information doesn't apply to your submission:

The statistical method used is described in the 'Analysis'. Randomisation test was used to determine statistical significance (*P* value) across the data, as it is well suited for smaller sample sizes. Source code of the randomisation test is made available via a direct link to GitHub repository (p. 33 in 'Analysis').
Each bar graph presented shows the values from individual patches. Source data are reported for summary plots in Figures 2, 5 and 6, with exact *P*-values between conditions in the Supplementary tables S1-3.

(For large datasets, or papers with a very large number of statistical tests, you may upload a single table file with tests, Ns, etc., with reference to sections in the manuscript.)

Group allocation

- Indicate how samples were allocated into experimental groups (in the case of clinical studies, please specify allocation to treatment method); if randomization was used, please also state if restricted randomization was applied
- Indicate if masking was used during group allocation, data collection and/or data analysis

Please outline where this information can be found within the submission (e.g., sections or figure legends), or explain why this information doesn't apply to your submission:

As described in the 'Analysis' section, the nature of the bis-MTS cross-linker or GluA2 mutant/construct was not masked during data acquisition or analysis. Testing of bis-MTS cross-linkers of different lengths was not done in any particular order and can be considered quasi-randomized. Post-hoc randomisation of the data is introduced via the randomisation test described above and in the 'Analysis' section in the manuscript. The field is yet to define the best way to perform masking and blinding during group allocation and data collection in patch-clamp experiments and we will continue to look for ways to securely implement that in our experimental routines.

Additional data files ("source data")

- We encourage you to upload relevant additional data files, such as numerical data that are represented as a graph in a figure, or as a summary table
- Where provided, these should be in the most useful format, and they can be uploaded as "Source data" files linked to a main figure or table

eLife Sciences Publications, Ltd is a limited liability non-profit non-stock corporation incorporated in the State of Delaware, USA, with company number 5030732, and is registered in the UK with company number FC030576 and branch number BR015634 at the address 1st Floor, 24 Hills Road, Cambridge CB2 1JP | August 2014

- Include model definition files including the full list of parameters used
- Include code used for data analysis (e.g., R, MatLab)
- Avoid stating that data files are "available upon request"

Please indicate the figures or tables for which source data files have been provided:

For each figure listed below, the following source data is provided as a Supplementary table: average values, the number of patches and standard error of the mean for each condition and *P* values across different conditions.
Figure 2D-E – source data in Supplementary Table S1
Figure 5B - source data in Supplementary Table S2
Figure 6C - source data in Supplementary Table S3

Source code for the software developed and used for the manuscript:
Figure 7 – code used for docking experiment is made available via a direct GitHub link (see section 'Analysis').
Code used to perform the randomisation test (used throughout the manuscript) is also made available via a direct GitHub link as described above and in the section 'Analysis'.
